# A highly selective mPGES-1 inhibitor to block abdominal aortic aneurysm progression in the angiotensin mouse model

**DOI:** 10.1038/s41598-024-57437-9

**Published:** 2024-03-23

**Authors:** Lauren M. Weaver, Madeline J. Stewart, Kai Ding, Charles D. Loftin, Fang Zheng, Chang-Guo Zhan

**Affiliations:** 1 Department of Pharmaceutical Sciences, College of Pharmacy, University of Kentucky, 789 South Limestone Street, Lexington, KY 40536 USA; 2 Molecular Modeling and Biopharmaceutical Center, College of Pharmacy, University of Kentucky, 789 South Limestone Street, Lexington, KY 40536 USA

**Keywords:** Aneurysm, Abdominal aortic aneurysm, Prostaglandin E2, mPGES-1 inhibitor, Anti-inflammation, Target validation, Molecular medicine

## Abstract

Abdominal aortic aneurysm (AAA) is a deadly, permanent ballooning of the aortic artery. Pharmacological and genetic studies have pointed to multiple proteins, including microsomal prostaglandin E_2_ synthase-1 (mPGES-1), as potentially promising targets. However, it remains unknown whether administration of an mPGES-1 inhibitor can effectively attenuate AAA progression in animal models. There are still no FDA-approved pharmacological treatments for AAA. Current research stresses the importance of both anti-inflammatory drug targets and rigor of translatability. Notably, mPGES-1 is an inducible enzyme responsible for overproduction of prostaglandin E_2_ (PGE_2_)—a well-known principal pro-inflammatory prostanoid. Here we demonstrate for the first time that a highly selective mPGES-1 inhibitor (UK4b) can completely block further growth of AAA in the *ApoE*−/− angiotensin (Ang)II mouse model. Our findings show promise for the use of a mPGES-1 inhibitor like UK4b as interventional treatment of AAA and its potential translation into the clinical setting.

## Introduction

Abdominal aortic aneurysm (AAA) is an irreversible dilation of the abdominal aorta. This is a diameter ≥ 3.0 cm reflecting 50% or greater growth in the infrarenal region^1^. AAA is a potentially lethal cardiovascular disease with no pharmacological treatment. Advanced age, smoking, and other cardiovascular diseases are also significant risk factors for AAA development^1,2^. Estimates put the pooled prevalence of AAA at 4.8% of the general population^3^. These aneurysms frequently go undiagnosed due to their asymptomatic nature. Risk of rupture is high at late stages and is the main complication of the disease. Mortality rates are inconsistently reported but average around 80% for ruptured aneurysms^3,4^. Unfortunately, there is no active pharmacological treatment for AAA. Options for patients remain as continued monitoring and surgical intervention for those that qualify. Lifestyle changes, such as smoking cessation, can be made in an attempt to abate disease progression. However, continued monitoring remains the only option and risky surgical intervention is only undertaken once patients meet disease progression criteria. This simple surveillance has shown mixed effects on patient quality of life^1,5,6^.

Three hallmark pathways of AAA etiology encompass vascular smooth muscle cell (VSMC) dysfunction, extracellular matrix (ECM) remodeling, and upregulated inflammation^7^. Progressive damage to VSMCs contribute to weakening of vascular tone through apoptosis and phenotypic switches. Elastin and collagen provide crucial support to the vessel wall but lose efficacy with negative remodeling. Inflammation remains a key mediator of AAA etiology^7^ with prominent inflammatory factors hypothesized to drive AAA formation and progression^7^.

Significant work has been conducted to evaluate novel drugs in treating AAA which we have previously reviewed^7^. Prior clinical trials have examined several drugs to halt AAA progression. However, the targets of these drugs have been primarily focused on one or two of the three core pathways. Doxycycline is a drug repurposed for AAA that initially held large promise. The tetracycline group of antibiotics to which doxycycline belongs, was found to have a novel mechanism of action as inhibitors of matrix metalloproteinases (MMPs), key regulators of the ECM and highly involved in AAA etiology. This activity was further exploited to modulate AAA progression through key ECM remodeling pathways that MMPs coordinate^8,9^. While it demonstrated great efficacy in preclinical models, this did not translate into the clinical trials conducted. Notably, very few of the preclinical studies backing clinically investigated compounds to treat AAA, such as doxycycline, have used an intervention-based treatment approach, instead focusing on a prevention model. Since the intervention in the main application of AAA treatment in the clinic, it is therefore crucial for investigational compounds to demonstrate efficacy in preventing further growth of AAA in animal models.

A central idea in the field suggests that inflammation is the main driver of AAA formation and progression, instigating the other two pathways as well, VSMC dysfunction and ECM remodeling. So far, only repurposed drugs have been clinically investigated for the treatment of AAA. Among these, a high number have targeted inflammatory factors implicated in the disease^7^. Canakinumab (ACZ885) is a human anti-IL-1β, pemirolast (CRD007) targets pro-inflammatory mast cells, mesenchymal stem cell therapy is possibly anti-inflammatory, metformin agonizes AMPK to regulate VSMC function and macrophages, and cyclosporin A inhibits T cell activation^10–17^. While inflammation is regarded as a critical target for AAA treatment, these drugs aimed to regulate inflammatory pathways but were not efficacious in the clinic. A crucial piece absent was supporting evidence demonstrating that the targeted inflammatory marker(s) also affected both VSMC and ECM pathways involved in AAA pathogenesis.

Great support is growing for microsomal prostaglandin E_2_ synthase-1 (mPGES-1) as a promising novel anti-inflammatory target for AAA^18–20^. The enzymatic product of mPGES-1, prostaglandin E_2_ (PGE_2_), is a prominent driver of inflammation. Together, the mPGES-1/PGE_2_/EP receptor axis has evidential data of both presence and function in AAA^21–23^. PGE_2_ contributes to AAA etiology by influencing not only inflammation, but also ECM degradation and VSMC dysfunction^21,23–25^. This makes it a potential target for AAA treatment as it hits all three key pathways. First, VSMC dysfunction: PGE_2_ increases reactive oxygen species and apoptosis in VSMCs and endothelial cells in AAA, causing damage to the arterial wall^20,23,26^. Second, ECM remodeling: PGE_2_ increases production of MMPs such as MMP-9, which is highly indicated in AAA, and decreases levels of tissue inhibitors of metalloproteinases (TIMPs)^24,27–30^. MMPs remodel the ECM in the aortic wall but overexpression causes excessive weakening of the structural support.

The mPGES-1/PGE_2_/EP receptor axis has been explored in animal models to develop a potential new AAA drug. Alterations of cyclooxygenase (COX), the enzyme directly up-stream of mPGES-1, such as inhibition and deletion/knockdown have evidenced effective in halting AAA formation in mice^31–33^. Inhibition and knockdown of PGE_2_’s EP_4_ receptor have also shown significant efficacy in preventing formation^19,25,30,34^. Finally, mPGES-1 knockout mice showed low rates of angiotensin II (AngII)-induced AAA formation^24^. Protein mPGES-1 is the favorable target of the three due to the severe side effects of COX-2 inhibitors and the role of EP_4_ expression in bone marrow-derived cells in protection against AAA formation in mice^35^.

To our best knowledge, we report here the first demonstration of a highly selective mPGES-1 inhibitor to effectively treat AAA progression in an animal model of AAA. Overcoming prior translational hurdles in the field, we have previously developed novel small-molecule mPGES-1 inhibitors potent against both mouse and human enzymes through computational design fitting the conserved regions in the active site^36^. Our lead compound UK4b is highly selective over COX-1 and COX-2 and is a potent inhibitor of both human (IC_50_ = 33 nM) and mouse (IC_50_ = 157 nM) mPGES-1^36^. Our most recent work has demonstrated significant anti-inflammatory and analgesic properties of UK4b by controlling PGE_2_ overproduction^37^.

In the present study we examined the efficacy of selective mPGES-1 inhibition in halting further progression of AAA in the AngII mouse model. We also report here for the first time a comparative study on the starting age of male *ApoE*−/− mice in the AngII model. Our studies herein demonstrate efficacy of highly selective mPGES-1 inhibition in halting further growth of an established AAA in these mice.

## Results

### Starting age of mice affects AAA progression

Increased age is a significant risk factor for the development of AAA in humans^38^. Similarly, the incidence of AAA following AngII infusion has been shown to be increased in aged (18–20 months) nonhyperlipidemic C57 wild-type mice, as compared to younger mice (2–3 months). Initial studies using the AngII infusion model in hyperlipidemic mice used mice aged 6–11 months, whereas the majority of more recent studies have used hyperlipidemic mice approximately 10–12 weeks of age^39–41^. In order to determine the effect of age on AAA formation in hyperlipidemic mice, which have significantly increased responsiveness to AAA formation following AngII infusion, we compared the effect of age on AAA development in *ApoE*−/− mice.

**Figure 1:**
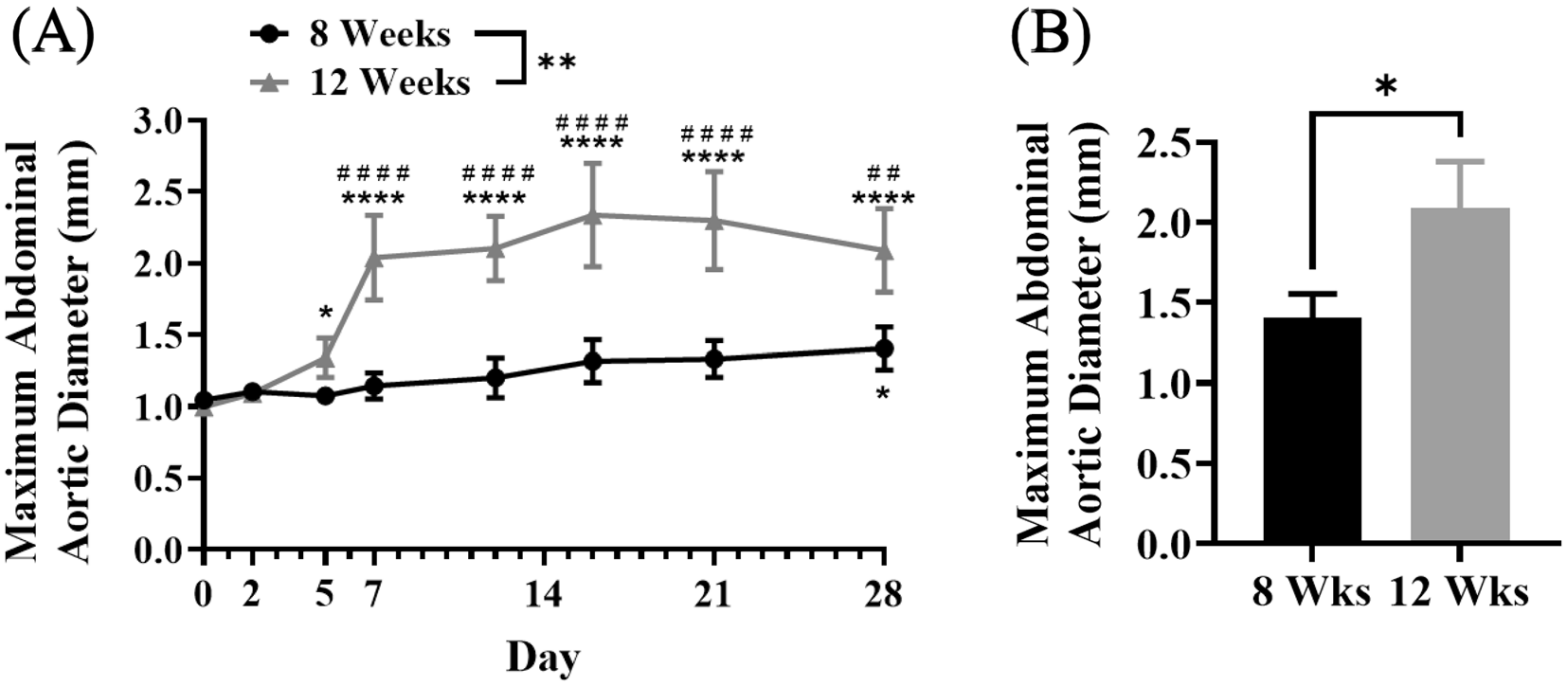
AAA progression in 8-week-old and 12-week-old mice. (**A**) Abdominal aortic diameter from day 0 to day 28 of AngII infusion. Mean ± SEM; *n* = 10 (8-weeks-old), *n* = 10 (12-weeks-old). *P < 0.05, ## **P < 0.01, #### **** *P* < 0.0001. Significance inter-group overall effect (*) by paired t-test (*P* = 0.0078). Significance intra-group versus day 0 (*) by two-way ANOVA. Significance inter-group each day (#) by two-way ANOVA. (**B**) Abdominal aortic diameter at day 28 of 8-week-old (8 weeks) and 12-week-old (12 weeks) mice. Mean ± SEM; *n* = 8 (8 weeks), *n* = 4 (12 weeks); * *P* < 0.05 (*P* = 0.0417) by unpaired t-test.

Herein we evaluated for the first time the effect of AngII infusion on AAA formation in *ApoE*−/− mice aged 8-weeks-old versus 12-weeks-old (Fig. 1). In vivo ultrasound over 28 days of AngII infusion (Fig. 1A) showed a statistically significant difference in growth rates between the two age groups (*P* = 0.0078). Significant rapid aortic dilation was observed by day 5 in the 12-week group (*P* = 0.0411) while the 8-week group showed slow progression, only reaching significant growth from day 0 by day 28 (*P* = 0.0315). The average maximum abdominal aortic diameter was significantly different between the groups from day 7 onwards. Overall, these data demonstrate a disparity in growth patterns and AAA formation between the two ages. On day 28, the final day of AngII infusion, there was a statistically significant difference (*P* = 0.0417) between the final maximum abdominal aortic diameter of the two groups (Fig. 1B; 8 weeks = 1.405 mm, 12 weeks = 2.088 mm).

Overall survival rates differed between the two groups. At day 28, 80% (8/10) of the 8-week-old mice had survived while half of that, 40% (4/10) of the 12-week-old mice had survived. AAA formation rates were significantly different between the two groups as well (*P* = 0.0003, Fisher’s exact test). At day 7, 10% (1/10) of the 8-week-old mice had an AAA while 75% (3/4) of the surviving 12-week-old mice had an AAA. At day 28, 50% (4/8) of the 8-week-old mice had an AAA while 100% (4/4) of the 12-week-old mice had an AAA. It appeared that lethal rupture usually occurred within the first week for the 12-week-old mice while in the 8-week-old mice this occurred after.

### mPGES-1 inhibitor interventional treatment halts further AAA progression

**Figure 2:**
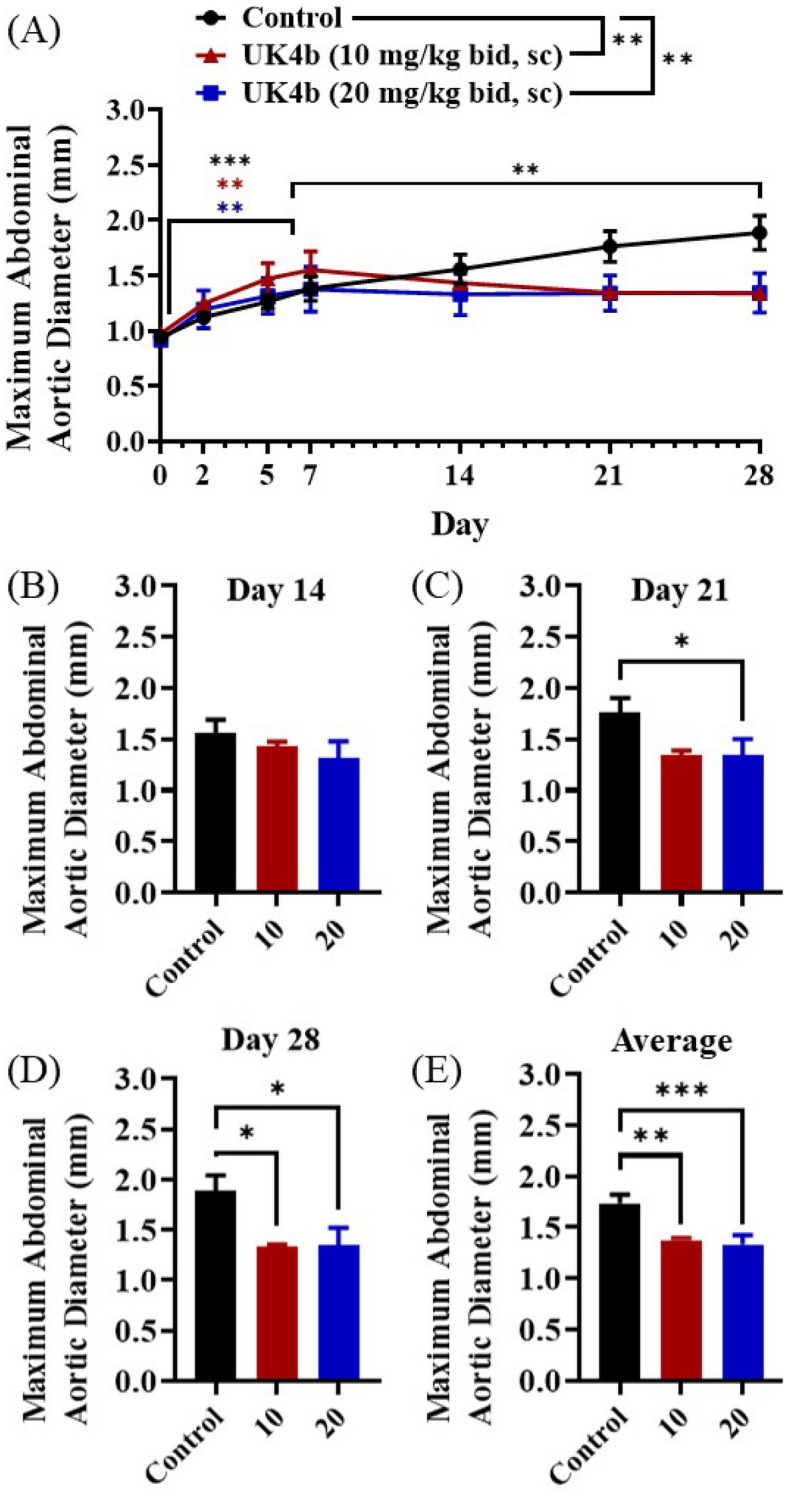
AAA progression in AngII only control mice and UK4b treated mice with administration started day 7. (**A**) Abdominal aortic diameter from day 0 to day 28 of AngII infusion. Mean ± SEM; *n* = 14 for control group in which one mouse died before day 7 and one mouse died after day 7, *n* = 6 for the 10 mg/kg group which one mouse died before day 7, *n* = 8 for the 20 mg/kg group in which one mouse died before day 7; *P < 0.05, **P < 0.01. Significance inter-group overall by one-way ANOVA (*P* = 0.0045 [control v 10], *P* = 0.0029 [control v 20], *P* = 0.6860 [10 v 20). Significance intra-group day 0 versus day 7 by two-way ANOVA (*P* = 0.0002 [control], *P* = 0.0018 [10], *P* = 0.0047 [20]). Significance intra-group day 7 versus day 28 by two-way ANOVA (*P* = 0.0046 [control], *P* = 0.4443 [10], *P* = 0.8842 [20]). (**B**) Abdominal aortic diameter on day 14. Mean ± SEM; *n* = 12 (control), *n* = 5 (10 mg/kg), *n* = 8 (20 mg/kg). (**C**) Abdominal aortic diameter on day 21. Mean ± SEM; *n* = 12 (control), *n* = 5 (10 mg/kg), *n* = 7 (20 mg/kg); *P < 0.05 (*P* = 0.0441 [control v 20]) by one-way ANOVA. (**D**) Abdominal aortic diameter on day 28. Mean ± SEM; *n* = 12 (control), *n* = 5 (10 mg/kg), *n* = 7 (20 mg/kg); *P < 0.05 (*P* = 0.0361 [control v 10], *P* = 0.0213 [control v 20]) by one-way ANOVA. (**E**) Average abdominal aortic diameter from day 14 through day 28. Mean ± SEM; *n* = 12 (control), *n* = 5 (10 mg/kg), *n* = 7 (20 mg/kg); **P < 0.01, ***P < 0.001 (*P* = 0.0069 [control v 10], *P* = 0.0008 [control v 20]) by one-way ANOVA.

Given that AAA was the most severe in the 12-week-old ApoE−/− mice, these were then used for our model. Rapid aortic dilation occurs during the first 7 days of AngII infusion and then is slower from day 7 to day 28^40,41^. Because of this, we chose to use day 7 for the initiation of our experimental drug intervention. To assess the capability of UK4b to halt further growth of AAA in the AngII mouse model, we infused these mice with AngII (1000 ng × kg^−1^ × min^−1^) for 28 days (Fig. 2). The two treatment groups started subcutaneous administration of UK4b twice a day at either 10 mg/kg (“10”) or 20 mg/kg (“20”) on day 7 through day 28 (Fig. 2A). A statistically significant difference was seen in overall AAA progression between the two treatment groups and the control group (Fig. 2A; *P* = 0.0045 [10 v. control], *P* = 0.0029 [20 v. control]). All three groups had significant growth in diameter by day 7 (*P* = 0.0002 [control],* P* = 0.0018 [10],* P* = 0.0047 [20]). There were no significant differences in diameter among the three groups on day 7.

UK4b treatment was started on day 7. On day 14 (7 days after UK4b treatment), the differences in diameter between the UK4b treatment groups and control were non-significant (Fig. 2B; control = 1.558 mm, 10 mg/kg group = 1.434 mm, 20 mg/kg group = 1.314 mm). On day 21, the average diameter in the 20 mg/kg UK4b group (1.341 mm) was significantly lower than that in the control group (1.764 mm), but the difference in diameter between the 10 mg/kg mg/kg and control groups was still insignificant (Fig. 2C). On day 28, the average diameters in both UK4b treatment groups were significantly shorter than that in the control group (Fig. 2D; control = 1.887 mm, 10 mg/kg group = 1.340 mm, 20 mg/kg group = 1.343 mm). Figure 2E shows the average diameter from day 14 to day 28 for each group. There was no significant difference in the average diameter between the two treatment groups on day 28, because the dose of 10 mg/kg UK4b has already reached the maximum possible efficacy of the mPGES-1 inhibition. So, it is understandable that starting from the dose of 10 mg/kg, further increasing the UK4b dose would not decrease the diameter further. The concept of reaching the maximum possible efficacy of the mPGES-1 inhibition is also supported by the following observation: There was no further AAA growth observed beyond day 7 in both UK4b-treated groups, while the control group exhibited further disease progression with significant continued aortic dilation (*P* = 0.4443 [10 mg/kg], *P* = 0.8842 [20 mg/kg],* P* = 0.0046 [control]).

Of note, there was a difference in the expected mortality rate of the mice in the study. In the age-related study reported above, there was a 60% mortality rate (6/10) in the 12-week-old mice. The 12-week-old control mice in this experiment had a 14% mortality rate (2/14) which is lower than anticipated based on the previous cohort. This unexpected result may potentially be due to variation in manufacturing batches of AngII or handling of the AngII when stored.

Overall, the 20 mg/kg treatment started to show significant efficacy on day 21, and the 10 mg/kg treatment started to show significant efficacy on day 28, suggesting the dose-dependent response to the UK4b treatment. However, since both the treatment groups have reached the maximum efficacy on day 28, our further analyses below will only consist of two groups, control and UK4b treatment (both doses merged).

Within the 2 control mice died, one died before day 7 and the other died after day 7. Compared to the control group, 2 out of 14 UK4-treated mice in (one in the 10 mg/kg UK4b group and one in the 20 mg/kg UK4b group) died before day 7; no animal died after the UK4b treatment started.

Additionally, we also analyzed the AAA incidence in both the control and UK4b treatment groups. As is known, AAA is a focal dilation in the infrarenal abdominal aorta with a diameter increased by ≥ 50%^42^. Using this criterion (≥ 50%), the AAA incidence data on days 7 and 28 are summarized in Table 1. Listed also in Table 1 are the AAA incidence data on days 7 and 28 using different criteria (≥ 30% and ≥ 70%). As seen in Table 1, no matter which one of the criteria is used, the AAA incidence always increased in the control mice, but always decreased in the UK4-treated mice, from day 7 to day 28.

**Table 1:** AAA incidence in control and UK4b-treated mice.

AAA incidence criterion (% increase in the diameter)^*a*^	Control mice^*b*^			UK4b-treated mice		
	Day 7 incidence	Day 28 incidence	Day 7 → 28 increase	Day 7 incidence	Day 28 incidence	Day 7 → 28 increase
≥ 30%	7/13	10/13	3	9/12	8/12	− 1
≥ 50%	5/13	10/13	5	5/12	2/12	− 3
≥ 70%	4/13	10/13	6	3/12	1/12	− 2

^a^Percentage increase in the diameter compared to the baseline diameter measured on day 0.

^b^One mouse (with an 83% increased on day 7) died after day 7 and was considered as AAA incidence on both days 7 and 28.

**Figure 3:**
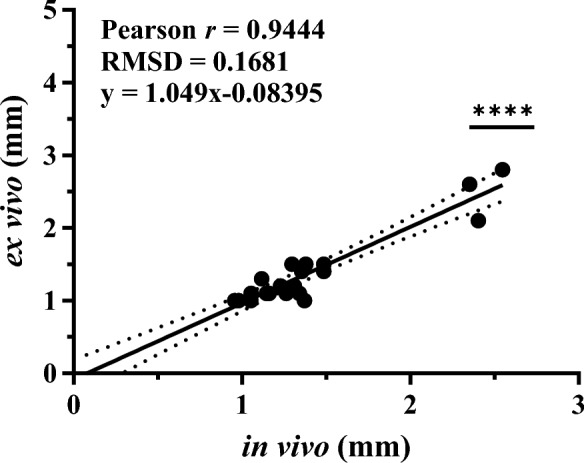
Correlation of final abdominal aortic diameter measurements by in vivo ultrasonography and ex vivo tissue for each mouse (all groups). Linear regression graphed with 95% CI. *n* = 20; ****P < 0.0001 by Pearson r correlation and linear regression analysis.

To validate the use of ultrasound to measure in vivo maximal internal abdominal aortic diameters, measurements of the day 28 in vivo ultrasound and ex vivo maximal external abdominal aortic diameters were compared for all animals (Fig. 3). There was a significant correlation between the in vivo and ex vivo measurements (Pearson *r* = 0.9444, *P* < 0.0001). A linear regression analysis exhibited excellent prediction and association of the values (*R*^2^ = 0.8919).

### UK4b attenuates AAA progression in relation to key biomarkers

**Figure 4:**
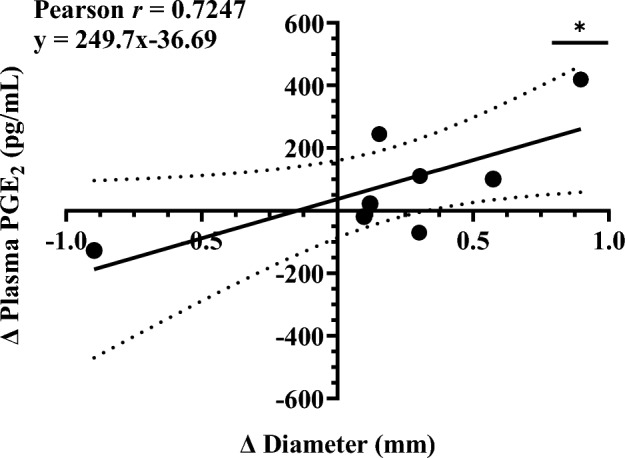
The change in maximal abdominal aortic diameter versus the change in plasma PGE_2_ between day 0–7 and day 7–28 (all groups). Linear regression graphed with 95% CI. *n* = 8; *P < 0.05 (*P* = 0.042) by Pearson r correlation and linear regression analysis.

We examined two biomarkers to better understand the downstream effects of mPGES-1 inhibition in relation to AAA progression. The first, PGE_2_, is the enzymatic product of mPGES-1 and a key inflammatory mediator that has been implicated in AAA pathogenesis^7,20,23,36,43–46^. We measured plasma PGE_2_ concentrations in study mice from all groups on days 0, 7, and 28 (Fig. 4). The changes in concentrations were calculated between two intervals, days 0–7 and days 7–28. This was then plotted against the change in maximum abdominal aortic diameter for the same time interval so that each point is a change in plasma PGE_2_ concentration versus the change in diameter between days 0 and 7 or days 7–28 for a mouse. There was a statistically significant correlation (*P* = 0.042) between the two variables (Pearson *r* = 0.7247). Therefore, the increase in AAA size is associated with an increase in plasma PGE_2_ concentration, which suggests the PGE_2_ levels may function as a key biomarker during AAA progression.

The second inflammatory biomarker we analyzed was plasma IL-1α. Previous studies suggest that IL-1α is a cytokine is significantly involved in AAA pathogenesis^47–49^. The inhibition of IL-1α has been shown to prevent AAA formation in mice, and surgical repair of AAA in humans reduces serum IL-1α concentrations^47–49^. Therefore, we measured plasma concentrations of IL-1α in control and UK4b treated mice on days 0, 7, and 28.

**Figure 5:**
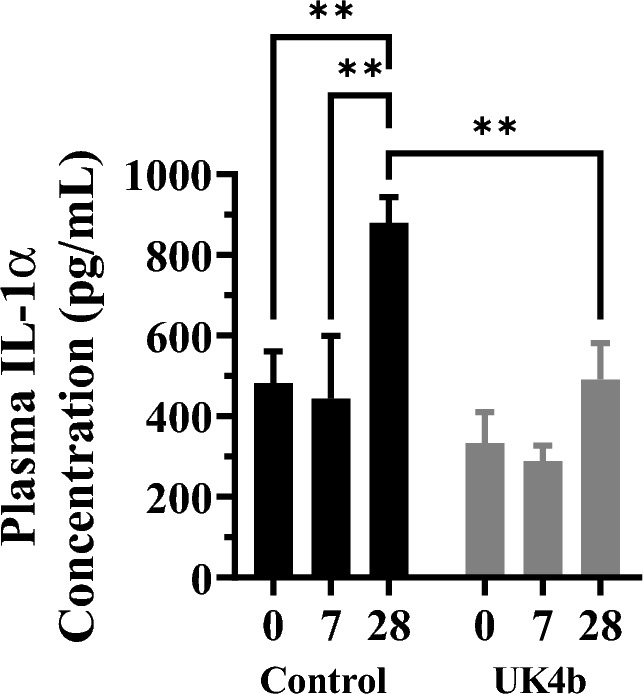
Plasma concentration of IL-1α at day 0, 7, and 28 in control and drug treated mice. Mean ± SEM; *n* = 9 (control day 0), *n* = 5 (control day 7), *n* = 8 (control day 28), *n* = 7 (UK4b day 0), *n* = 8 (UK4b day 7), *n* = 13 (UK4b day 28); **P < 0.01 (*P* = 0.0021 [control day 0 v day 28], *P* = 0.0038 [control day 7 v day 28], *P* = 0.0012 [UK4b v control]) by two-way ANOVA.

As shown in Fig. 5, UK4b treatment resulted in statistically significant differences in IL-1α as compared to controls. In the control group, an increase was observed on day 0 and day 7 *versus* day 28 timepoints (*P* = 0.0021, *P* = 0.0038). This increase was not observed in the UK4b treatment groups. Additionally, there was a significant difference between the two groups on day 28 (*P* = 0.0012). UK4b administration prevented increased expression of IL-1α at day 28. These findings indicate that increased plasma IL-1α correlates with AAA progression. Furthermore, our findings suggest that the effectiveness of mPGES-1 inhibition with UK4b in the elimination of AAA progression is associated with the attenuation of IL-1α plasma levels.

### Histological analysis of abdominal aortas

**Figure 6:**
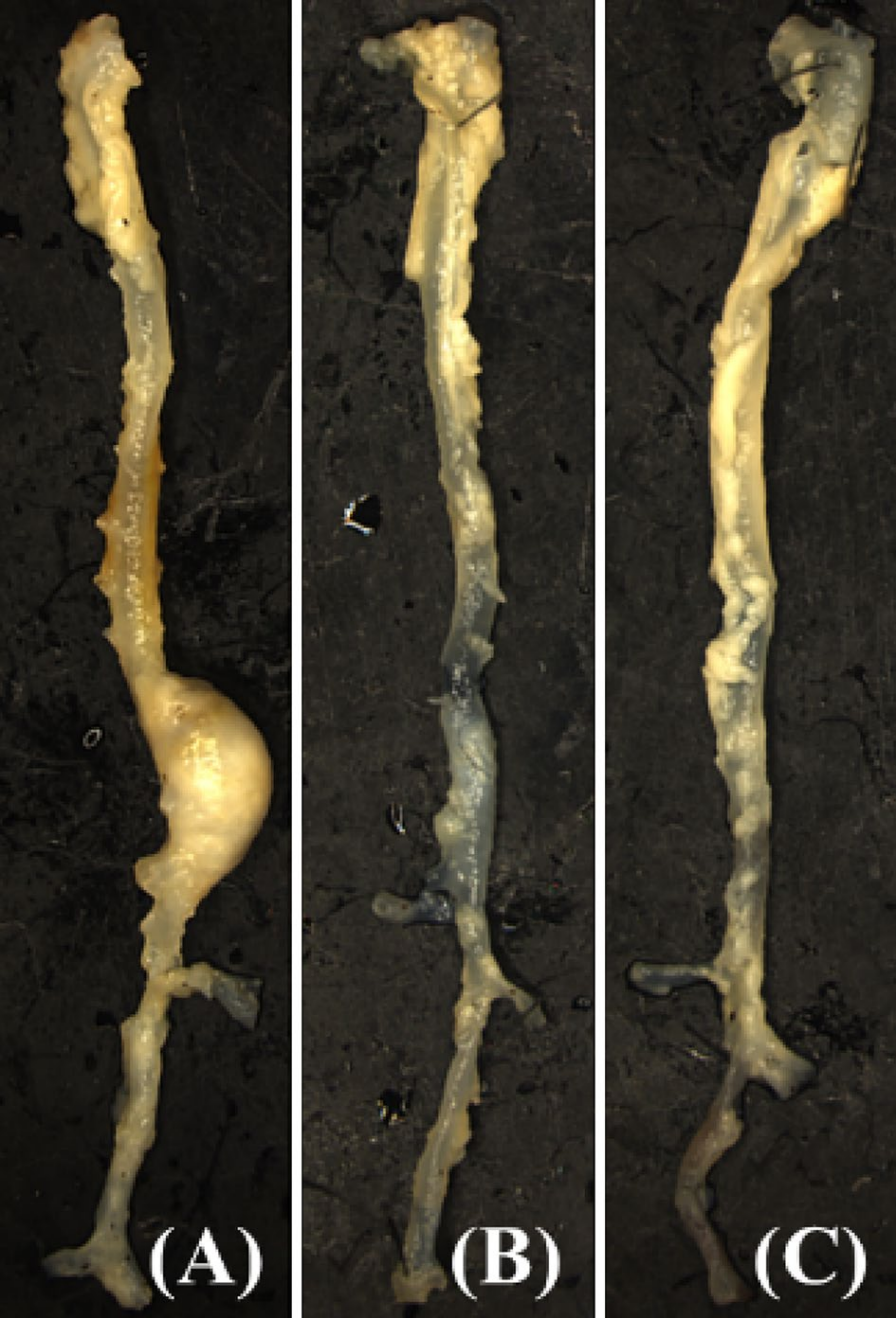
Representative ex vivo* a*ortic tissues on day 28 in the three groups. Tissues were chosen as those that best represented the consistent pathology seen in each group. (**A**) Control, (**B**) UK4b 10 mg/kg, (**C**) UK4b 20 mg/kg.

Once mice were sacrificed on day 28, aortic tissues were collected for histological analysis. Representative ex vivo aortas of the control, 10 mg/kg treatment, and 20 mg/kg treatment groups are shown in Fig. 6. The control animal tissue (Fig. 6A) has a noticeably large sized aneurysm in the abdominal aortic region, above the renal arteries. Suprarenal AAA is a distinct feature of the AngII mouse model. In contrast to infrarenal AAA in humans, the AngII mouse model seldom involves the aortic region where the renal arteries branch^7^. Both of the tissues from UK4b treated mice do not have the large dilations or notable AAA formation as occurs in the untreated control group (Fig. 6B,C). Therefore, our ex vivo observations of abdominal aortas support our in vivo analysis of increased AAA formation in the untreated control mice.

**Figure 7:**
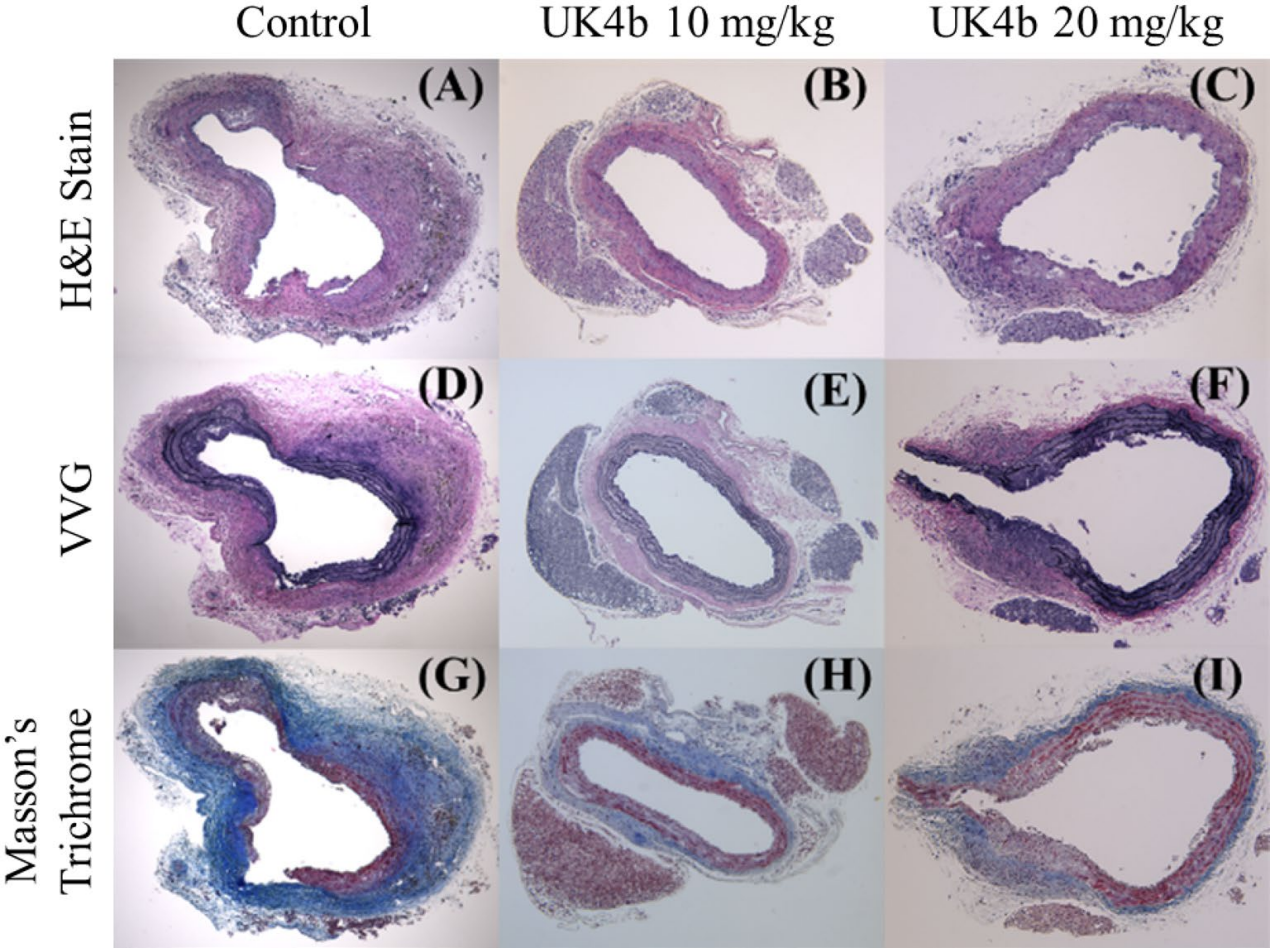
Tissue histology of mouse abdominal aorta at the maximum width: (**A**–**C**) H&E, (**D**–**F**) VVG, and (**G**–**I**) Masson’s Trichrome. Treatments: (**A**, **D**, **G**) control, (**B**, **E**, **H**) UK4b 10 mg/kg, and (**C**, **F**, **I**) UK4b 20 mg/kg.

After ex vivo measurements were taken, tissues were sent for histological staining as shown in Fig. 7. Tissues from each of the 3 groups, control (Fig. 7A,D,G), UK4b 10 mg/kg (Fig. 7B,E,H), and UK4b 20 mg/kg (Fig. 7C,F,I) were sectioned at 5 µm from the widest part of the aneurysm (or if none, right above the renal branches), mounted on slides, and stained with H&E (Fig. 7A–C), Verhoeff–Van Gieson (VVG; Fig. 7D–F) for elastin, and Masson’s Trichrome (Fig. 7G–I) for collagen.

H&E staining revealed that control mice (Fig. 7A**)** had significant thickening of the arterial wall with visible disruption to the layers. This was not apparent in the tissues collected from the UK4b treated mice (Fig. 7B,C), which maintained most of their structuring with minimal inflammation observed. VVG staining for elastin showed significant straightening of the normally undulating elastin fibers in the control tissue (Fig. 7D) indicating disruption to the elastin layers of the aortic wall. This was not as observable in the tissues from the UK4b 10 mg/kg (Fig. 7E) and UK4b 20 mg/kg treated mice (Fig. 7F). Masson’s Trichrome staining reflected significant thickening of the media in the tissue of the control mice (Fig. 7G) with disruption of the collagen layers. Tissues from the UK4b treated mice showed minor thickening and consistent maintenance of congruent layers.

## Discussion

Currently available research has indicated that PGE_2_ and mPGES-1 participate in AAA initiation or progression. Prior publications have shown that human aneurysmal tissue has significantly upregulated mPGES-1 axis, expressing high levels of PGE_2_, mPGES-1, and the EP_4_ receptor^21–23^. In addition, studies using animal models of AAA have shown reduced AAA initiation or progression following pharmacological inhibition or genetic deletion of COX-2, mPGES-1 genetic deletion, and EP_4_ receptor antagonism or genetic deletion^23–25,30–34,50^. Therefore, although COX-2 and the EP_4_ receptor have been identified as a potential pharmacological target for the future treatment of AAA, no such evidence has previously been presented for mPGES-1. To the best of our knowledge, this is the first demonstration of highly selective mPGES-1 inhibition to halt progression of AAA in an animal model of the disease. Herein, we show the effectiveness of mPGES-1 inhibition for disrupting AAA progression when treatment begins following initiation of the disease in the AngII-infused *ApoE*−/− mouse model.

There is no previous publication that has examined the influence of the age of the *ApoE*−/− mice age on AAA progression using the AngII infusion model. Here we have shown for the first time that the age of *ApoE*−/− mice significantly affects AAA formation and progression rates during the AngII infusion. The 12-week-old mice developed AAA at a significantly greater rate than 8-week-old mice over the 28 days of AngII infusion. The rate of AAA formation and progression was significantly stunted in the 8-week-old *ApoE*−/−. Overall, the younger mice had fewer aneurysms and were smaller when present. These findings provide supportive justification for the use of 12-week-old mice in our AAA studies.

Our primary goal of this study was to determine whether selective mPGES-1 inhibition would effectively prevent further AAA progression after initiation of the disease. Although there are numerous studies that have identified potential targets that are effective for preventing AAA formation in animal models, there are relatively few studies that have identified a target that is effective for intervention after the initiation of AAA formation. Intervention-based treatment studies are crucial for the eventual identification of pharmacological treatments to be utilized in clinical trials. In the current study, we used in vivo ultrasound imaging of the internal diameter of the abdominal aorta to determine that aggressive aneurysmal dilation occurs in the first week of the AngII infusion, which is then followed by less aggressive expansion during days 7–28 of the AngII infusion. Our findings determined that rather than resulting in a partial slowing of disease progression, starting mPGES-1 inhibitor treatment on day 7 of the AngII infusion resulted in a complete disruption of further AAA progression through completion of the 28-day AngII infusion. These positive results of the mPGES-1 inhibitor are encouraging for the future translational potential of a pharmacologic interventional treatment for AAA.

Our current findings align with our previously published work demonstrating analgesic and anti-inflammatory effects of UK4b through regulation of PGE_2_^37,46^. Similarly, our current study shows that PGE_2_ concentrations were also observed to be significantly decreased in UK4b treated mice. Furthermore, we found a significant correlation between changes in plasma PGE_2_ concentration and changes in AAA diameter. In both control and UK4b treated mice, there was a positive relationship between attenuation of aortic dilation and prevention of PGE_2_ production. One consideration for this analysis is the instability of PGE_2_ in samples. PGE_2_ has a short half-life in blood, often limiting quantification. With this limitation in mind, the absolute PGE_2_ levels in blood are expected to be underestimated. Nevertheless, in consideration of the limitation, we collected blood samples with indomethacin and assured consistent treatment of all samples to reduce potential variability. Study findings demonstrated relative differences between treatment groups which afforded insights into the mechanisms of AAA treatment.

Interestingly, we found that the administration of UK4b prevented an overexpression of serum IL-1α concentrations, as compared to controls. A plethora of cytokines are known to be involved in AAA pathogenesis but less data are present regarding IL-1α. Previous studies in human AAA patients have reported a significant increase in blood IL-1α levels, as compared to those without AAA^48,51,52^.

Animal data are controversial on the effect of IL-1α on AAA in mice, leaving inconsistent conclusions on the relationship. Kawasaki Disease (KD), a childhood disease of systemic vasculitis, is often associated with AAA. Wakita et al. examined the role of IL-1α on the development of AAA in a mouse model of KD^53^. The homozygous knockout of either IL-1α or the IL-1α receptor prevented formation of AAA in the model. It was also demonstrated that treatment with an anti-IL-1α neutralizing antibody inhibited AAA formation. Alternatively, another study using the elastase model of AAA observed that IL-1α deficient C57BL/6 mice had significantly larger AAA diameters compared to the wild-type controls^54^. Furthermore, administration of an anti-IL-1α neutralizing antibody exacerbated AAA formation in the study.

Our findings reported here provide evidence to conclude a potential relationship between IL-1α levels and AAA in mice. We have found that interventional treatment with the mPGES-1 inhibitor UK4b prevented a significant increase in the serum IL-1α levels of AngII-infused *ApoE*−/− mice, which was observed in concert with attenuation of AAA progression. This is notably similar to the clinical report that surgical intervention for aneurysm repair significantly reduced patient IL-1α serum levels^48^. In the elastase induced AAA model, IL-1α-deficient C57BL/6 mice had larger AAA sizes, as compared to wild-type controls. In comparison, our mPGES-1 inhibitor prevented the significant upregulation of IL-1α, thereby maintaining basal levels of the cytokine expression and halting further aortic dilation.

Previous studies have established a correlation between PGE_2_ and other inflammatory cytokines. IL-1α and PGE_2_ are both involved in AAA, but no link has been established between the two until our current findings. Our data bring the first supportive evidence that an mPGES-1 inhibitor may indirectly regulate overexpression of IL-1α via PGE_2_ in AAA. Herein we demonstrate the effective inhibition of IL-1α overproduction via the regulation of PGE_2_ levels. To our knowledge, this is the first demonstration of an association between these two inflammatory mediators during AAA development in mice.

Traditional non-steroidal anti-inflammatory drugs (NSAIDs) target inflammation by inhibiting COX to reduce production of prostanoids, primarily PGE_2_. However, NSAID use is strongly linked to cardiovascular side effects. This is believed to be associated with the decrease in cardioprotective prostacyclin (PGI_2_) production. As an alternative, downstream selective mPGES-1 inhibition reduces proinflammatory PGE_2_ without also reducing cardioprotective PGI_2_. Previous work has reported that deletion or inhibition of mPGES-1 was not observed to decrease PGI_2_ in vessels, unlike COX-2 inhibition which did; inhibition of mPGES-1 was even observed to reduce human vascular tone by increasing PGI_2_^55–58^. Similarly, levels of asymmetric dimethylarginine (ADMA), an established cardiotoxic biomarker, are not affected by mPGES-1 modulation yet increased with loss of COX-2^57^. Selective inhibition of mPGES-1 may be a safer alternative to COX inhibition. Considering these previously reported mechanistic insights, the cardioprotective effects seen with the mPGES-1 inhibition, like those reported here, could be a collective result of co-modulation of multiple biomarkers such as PGI_2_ and ADMA, in addition to direct modulation of PGE_2_ itself.

In conclusion, we have displayed herein the effectiveness of highly selective mPGES-1 inhibition in completely inhibiting further AAA progression in AngII-infused *ApoE*−/− mice. Supportive data also support the interrelationship between concomitant prevention of PGE_2_ and IL-1α overproduction. Selective inhibition of mPGES-1 is a promising future drug target to treat inflammatory diseases such as AAA. There is exciting potential for future studies to support clinical trials to evaluate the efficacy of mPGES-1 inhibition in humans. The body of evidence herein supports the potential of an mPGES-1 inhibitor for the treatment of AAA, and the implication of PGE_2_ as a key biomarker in disease etiology.

## Materials and methods

### Materials and instrumentation

The UK4b compound used was synthesized as previously discussed^36^. All animal experiments described here were performed with the approval of the University of Kentucky Institutional Animal Care and Use Committee (IACUC #2019-3373) and in accordance with the Guide for the Care and Use of Laboratory Animals from National Institutes of Health/Office of Laboratory Animal Welfare (NIH/OLAW), and were in fact also consistent with the ARRIVE (Animal Research: Reporting of In Vivo Experiments) guidelines (https://arriveguidelines.org). Animals were housed in the University of Kentucky DLAR facilities inside clean cages with ad libitum access to normal chow and water. Buprenorphine, ketamine, and xylazine were purchased from Covetrus (Dublin, Ohio, USA). Angiotensin II (AngII) was purchased from VWR (Radnor, PA, USA). ALZET Osmotic Pumps (model 2004) were purchased from Braintree Scientific (Braintree, MA, USA).

In vivo ultrasound imaging was done using a Vevo 3100 Ultrasound (Fujifilm VisualSonics, Inc.) and data analyzed using VevoLAB software (Fujifilm Visualsonics, Inc.) whose use was provided by the University of Kentucky Cardiovascular Research Priority Area. PGE_2_ was measured using the commercially available enzyme linked immunosorbent assay (ELISA) kit (catalog #514531) from Cayman Chemical (Ann Arbor, MI).

### AngiotensinII-infused mouse model of AAA

Male apolipoprotein E knockout (*ApoE*−/−) mice (JAX strain #002052) were ordered from Jackson Laboratories (Bar Harbor, Maine, USA). ALZET Osmotic Pumps (model 2004) were filled with AngII (1000 ng × kg^−1^ × min^−1^) and implanted subcutaneously for 28 days as described^41^.

#### Age progression study

There were two groups of mice representing their ages at the start of AngII infusion (day 0), 8-weeks-old and 12-weeks-old. All mice were infused with AngII (1000 ng × kg^−1^ × min^−1^) for 28 days with in vivo measurements taken by ultrasound on days 0, 2, 5, 7, 12, 16, 21, and 28. Mice were sacrificed on day 28 after final measurements.

#### UK4b treatment study

On day 0 before the first in vivo measurement, 12-week-old mice were divided into three groups: control (AngII only), treatment with UK4b 10 mg/kg, or UK4b 20 mg/kg. All mice were infused with AngII (1000 ng × kg^−1^ × min^−1^) for 28 days (ALZET pump model 2004). Starting on day 7, treatment mice were administered UK4b suspended in vegetable oil BID SC at either 10 mg/kg or 20 mg/kg. In vivo measurements were taken by ultrasound on days 0, 2, 5, 7, 14, 21, and 28. Mice were sacrificed on day 28 after final in vivo measurements.

### In vivo ultrasound measurement

The maximal abdominal aortic diameter of mice was measured in vivo over the course of the study by Vevo 3100 Ultrasound (Fujifilm VisualSonics, Inc.). Mice were anesthetized with isoflurane and taped supine on a warming table. Depilatory cream was used to remove hair on the abdomen and thoroughly wiped away with cotton swabs and baby wipes. Warm ultrasound transducer gel was placed on the abdomen prior to transducer placement perpendicular to the aorta. The right renal artery branch was used as a landmark to further identify the maximum abdominal aortic diameter by ultrasound. One hundred frame cine loops were captured for data analysis.

Ultrasound data was analyzed afterwards using VevoLAB software (Fujifilm Visualsonics, Inc.). After reviewing the cine loops, three separate frames were selected from three individual periods of diastole in the cardiac cycle (the aorta is at its smallest diameter). On each frame, the two longest diameter lengths were measured. The larger of these two measurements was then averaged together with that of the other two frames to give the maximal abdominal aortic diameter of that mouse at that timepoint. An aneurysm was defined on ultrasound as an internal abdominal aortic diameter of ≥ 50% greater.

### Analysis of prostaglandin E_2_

Blood samples were collected from mice on days 0, 7, 28, and 77 (if applicable) of the study via the saphenous vein. Blood was collected in an epi-tube with EDTA and indomethacin and spun down at 13.1 min^-1^. The plasma was then removed and frozen until analysis. PGE_2_ was quantified in plasma using Cayman’s ELISA kit (catalog #514531) per manufacturer’s instructions.

### Tissue histology

On the last day of the study, animals were sacrificed, and the aorta removed (heart inclusive down to the iliac branch) for analysis. Tissues were measured (reported ex vivo measurements) and photographed, then fixed in 4% paraformaldehyde before being washed and transferred into PBS. Samples were sent to the University of Kentucky COCVD Pathology Research Core for processing and histological staining. Briefly, tissues were embedded in paraffin, sectioned at 5 µm, and mounted on slides before staining. Stains used were hematoxylin and eosin (H&E), Masson’s Trichrome for collagen, and VVG (Verhoeff Van Gieson) for elastin.

### Cytokine analysis

Cytokines were measured in mouse plasma on days 0, 7, and 28 of AngII infusion via the saphenous vein. Plasma was shipped to Eve Technologies (Calgary, AB, Canada) on dry ice to be analyzed.

### Statistical analysis

Mean and SEM were calculated and displayed for measurements as indicated. Data were analyzed by student t-test, one-way analysis of variance (ANOVA), or two-way ANOVA. Correlation was calculated using the Pearson correlation coefficient with simple linear regression and 95% confidence interval bands. A Chi-squared or Fisher’s exact test was used to evaluate incidence rates. All statistical analyses and graphing were done with GraphPad Prism software (GraphPad Software, La Jolla, CA, USA). Statistical significance: *P < 0.05, **P < 0.01, ***P < 0.001, and ****P < 0.0001.

## Data Availability

The datasets used and/or analyzed during the current study are available from the corresponding author on reasonable request.
